# Central and peripheral fatigue development in the shoulder muscle with obesity during an isometric endurance task

**DOI:** 10.1186/s12891-017-1676-0

**Published:** 2017-07-21

**Authors:** Mojdeh Pajoutan, Mahboobeh Ghesmaty Sangachin, Lora A. Cavuoto

**Affiliations:** Industrial and Systems Engineering, University at Buffalo, 324 Bell Hall Buffalo, New York, 14260 USA

**Keywords:** Central fatigue, Peripheral fatigue, Shoulder muscle, Electrical stimulation, Obesity

## Abstract

**Background:**

Fatigue increases the likelihood of developing work-related musculoskeletal disorders and injury. Due to the physiological and neuromuscular changes that accompany obesity, it may alter the fatigue development mechanism and exacerbate injury risk. The upper extremities have the highest incidence rates for work-related musculoskeletal disorders. Therefore, the goals of this study were to investigate the effect of obesity on central vs. peripheral fatigue as well as on the physical signs of fatigue on the middle deltoid muscle.

**Methods:**

A measure of central activation ratio was used to quantify central fatigue by considering the increment in the torque output by superimposed twitch relative to its corresponding maximum voluntary contraction. For this purpose, electrical stimulation was delivered at the middle deltoid muscles of 22 non-obese (18 < body mass index (BMI) < 25 kg/m^2^) and 17 obese (30 < BMI < 40 kg/m^2^) individuals aged 18-32 years old. Participants completed superimposed maximum voluntary isometric contractions of shoulder abduction before and after a sustained isometric fatiguing task at either 30 or 60% of the muscle capacity. Differences in endurance time, torque fluctuation, torque loss, and muscle activity measured by an electromyography sensor were also investigated.

**Results:**

A greater reduction of voluntary activation of motor units (*p* = 0.001) with fatigue was observed for individuals who are obese. Contrary to the effect of obesity on central fatigue, a trend toward reduced peripheral fatigue (*p* = 0.06) was observed for the obese group compared to the non-obese group. On average, a 14% higher rate of torque loss per second was observed among individuals with obesity in comparison to non-obese participants.

**Conclusions:**

The observed greater contribution of central fatigue during the sustained endurance tasks suggests that among young healthy obese individuals, the faster fatigue development with obesity, commonly reported in the literature, is most likely due to the central elements rather than the peripheral factors. This finding has implications for fatigue prevention programs during sustained exertions and can help to develop training, work, and rest schedules considering obesity.

## Background

The growing prevalence of obesity worldwide (13% of the population) [1]; and in the USA (37.7% in 2014) [2] has resulted in negative consequences such as increased risk of work-related musculoskeletal disorders (WMSDs) [3], lost workdays, and related economic burden [4, 5]. Among WMSDs, injuries of the upper extremities had the highest incidence rate (32%), most frequently occurring at the hand (12.7%) and shoulder joint (8.2%) [6]. With increasing BMI, the chances of neck/shoulder injury claims increase [7] to the extent that workers with shoulder pain are twice as likely to have a BMI ≥ 29 kg/m^2^ [8]. In particular, abdominal obesity was identified as a significant factor affecting experience of shoulder joint pain during actively resisted movements such as shoulder abduction [9].

For individuals who are obese there was a longer arm movement time to complete rapid tasks [10] and a farther reach from the work area due to an increased abdominal circumference [11, 12]. In addition to the obesity-related changes in body part morphology, blood flow and oxygen supply to the muscle are decreased as a result of decreased capillary density [13, 14]. A higher proportion of fast-twitch type II fatigable muscle fibers is evident with obesity, and perfused fat in the muscle limits the muscle’s ability to contract due to interference with the muscle structure [15, 16]. Therefore, greater fatigue development is reported to accompany obesity. In agreement with this, shorter endurance times and higher rates of strength loss with obesity have been observed during shoulder flexion tasks [17, 18].

Fatigue interferes with force generation [19] and muscle motor control capabilities, increases the likelihood of WMSD development [20, 21], and decreases neural drive to the motor units [22]. Muscle fatigue might occur at the muscle (peripheral) or central nervous system (CNS) (central fatigue) levels. Understanding the obesity-related differences of central versus peripheral fatigue requires an examination of the force production pathway both at the neuromuscular junction and at the muscle level.

At neuromuscular junction, central fatigue can occur as a reduction in voluntary activation of motor neurons due to neural drive deficiency, signal propagation impairment, incomplete motor unit activation [23], and lack of motivation or pain tolerance [24]. Methods used to detect the central elements of fatigue vary. Due to the non-specifity of transcranial magnetic stimulation [25], electrical stimulation of motor nerves during voluntary contractions is commonly used [26]. An increased chance of signal propagation failure for type II muscle fibers [27] and a higher perceived postural stress reported with increased BMI [28] may imply a greater contribution of central fatigue for individuals who are obese. Supporting this theory, obesity-related reduction in central activation and neuromuscular control of the lower extremities were previously diagnosed by means of superimposing electrical stimulation (ES) signals [29–31]. With obesity manifested particularly in the lower extremities, the chronically imposed load of excess fat, is reported to have similar effects as weight training and alters fatigue development [32]. Further examinations of central fatigue with obesity in the upper extremities and, of particular interest in this study, on the middle deltoid muscle are necessary.

Peripheral fatigue, on the other hand, is a decline in the force generating capacity as a function of differences at the muscle level resulting from fat-free cross-sectional area [33], muscle contractile properties and intramuscular oxidative metabolism [34, 35], or impaired excitability or excitation-contraction coupling [36]. Peripheral fatigue was previously quantified by measuring the twitch response of an inactivated muscle to a single stimulus [37]. Reduction in the muscle twitch amplitude following a fatigue protocol was used as an indication of peripheral fatigue [38]. Physiological changes with obesity necessitate further research on the possible altered role of peripheral fatigue with obesity.

It remains unknown whether fatigue resistance of the deltoid muscle with obesity would be affected at the muscle- or neural-level. Therefore, the main objective of this study was to quantify obesity-related differences in the muscle fatigue mechanism in terms of central versus peripheral fatigue for the commonly used middle deltoid muscle. It was hypothesized that ES would determine an altered contribution of central and peripheral fatigue with obesity. A reduced muscle activation after fatigue was anticipated for individuals who are obese. Markers of fatigue such as decreased endurance time, torque fluctuation, torque loss and impaired or altered task performance have been previously reported to be impacted by obesity. An inverse relation between BMI and endurance time during sustained isometric contractions at 30% of maximum voluntary contractions (MVCs) was reported by Eksioglu [39]. Moreover, Cavuoto and Nussbaum [18] reported higher rates of strength loss and increases in discomfort among obese compared to non-obese adults. Similarly, in a lifting study, individuals with obesity changed their lifting mechanism when fatigued, by increasing their trunk transverse and sagittal posterior accelerations while the non-obese maintained theirs [40]. Therefore, as a secondary objective, the effect of obesity on general fatigue manifestation during the endurance task was studied. A reduced muscle functional capacity with obesity was hypothesized.

## Methods

### Participants

Thirty-nine young healthy individuals aged 18-32 years volunteered from the university and local communities to form two groups of 22 non-obese (18 < BMI < 25 kg/m^2^) and 17 obese (30 < BMI < 40 kg/m^2^) participants. The non-obese group consisted of 11 males and 11 females and the obese group included 11 males and six females. Detailed anthropometric and demographic information is provided in Table 1. The experiment was approved by the University at Buffalo Institutional Review Board and all participants provided written informed consent. To qualify for the experiment, participants were required to have no history of physical disorders at the shoulder joint. In addition, those who had extensive physical activities like heavy lifting, digging, aerobics or fast bicycling for more than 3 h per week were excluded from this experiment. Weight and body fat percentage (%BF) were measured using an electronic impedance scale (BC-568 Inner Scan, TANITA Corporation, Tokyo, Japan).

**Table 1:** Participants’ information presented as mean (SD)

	Normal (*n* = 22)	Obese (*n* = 17)
Age (yr)^*^	21.5 (2.3)	23.2 (3.5)
Body mass (kg)^*^	63.1 (9.4)	98.0 (10.4)
Stature (cm)^*^	168.3 (8.46)	173.1 (8.65)
BMI (kg/m^2^)^*^	22.2 (2.0)	32.7 (2.6)
Body fat (%)^*^	21.8 (7.3)	35.2 (6.8)
Fat free mass (kg)^*^	49.7 (10.3)	63.8 (11.4)
Waist circumference (cm)^*^	76.5 (5.8)	102.8 (8.6)
Hip circumference (cm)^*^	82.2 (5.8)	108.6 (8.3)
Waist to hip ratio^*^	0.93 (0.02)	0.95 (0.02)

*indicates a significant difference at *p* < 0.05 based on a *t*-test

### Experimental design

**Fig. 1:**
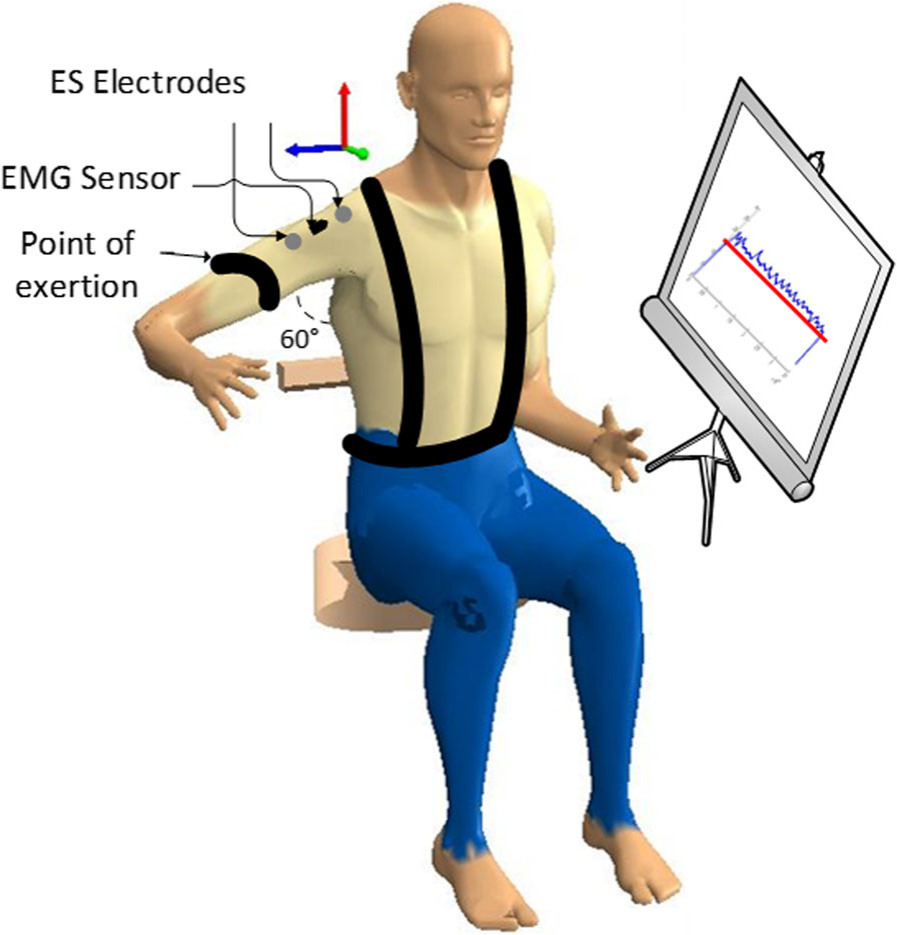
Experimental set-up simulated in 3DSSPP

Participants sat upright in an isokinetic dynamometer (Cybex Humac NORM, Ronkonkoma, NY, USA) chair with their torso strapped to the chair by shoulder and seat stabilizer belts. Their right shoulder was abducted at 60°, elbow flexed at 90° and hand faced downward in a neutral position with their feet in a footrest with knees flexed at 90°. This position of the arm falls within the middle of the range of motion of the shoulder abduction, as the prime recruiter of the middle deltoid [41], and is commonly used during activities of daily living. Pilot testing confirmed the suitability of this testing position in terms of maximum output while isolating middle deltoid as much as possible. The shoulder angle was confirmed with a goniometer. All participants completed the task with their right arm. A padded shoulder adaptor was attached ~10 cm down from the acromion process and firmly secured to detect even small movements but not too tight to occlude blood circulation. This attachment supported the weight of the upper arm. Visual feedback was provided on a monitor in front of the subject. Figure 1 illustrates the experimental setup schematically.

Two 3.2 cm diameter round surface ES electrodes were placed ~2.5 cm apart [42] longitudinally on the motor points of the middle deltoid. The cathode was placed above the anode for more effective results [43]. When needed, electrodes were trimmed to fit in the middle deltoid of each subject. An electromyography (EMG) mini sensor (Trigno Wireless, Delsys Systems, MA) was attached in between the ES electrodes on the middle deltoid muscle belly [44] to collect EMG signals at a collection frequency of 2048 Hz. The skin was shaved and cleaned prior to electrode placement. ES signals were set as single 70 ms supramaximal voltage electrical signal, delivered by a stimulus isolation unit and constant current unit connected in series (Grass Instruments S88 stimulator, SIU5 stimulus isolation unit, and CCU1 constant current unit, Natus Neurology, West Warwick, RI). For each individual, signal intensity and optimal location of the ES electrodes were examined by tracking the changes in EMG M-wave amplitude until it reached its maximum. This method has been used extensively in the literature to account for inter-individual variability of the motor point locations and pain thresholds (i.e., [35, 45–47]). Maximal tolerable current was found individually by progressively increasing the intensity by 10 mA, with a limit of 50 mA, until the M-wave amplitudes plateaued. A custom LabView program (version 13.0.0) was coded to drive the stimulator, alarm the participants to start and stop each subtask, and log the torque data for further analyses. Torque data were acquired at 1024 Hz rate and low-pass filtered using a fourth order Butterworth filter with a 4 Hz cutoff frequency.

After a short warm up including repeated shoulder abductions and adductions, the experiment started with an ES delivered at muscle rest (ES_0_) while participants were instructed to sit relaxed and keep their arm in the described posture without exerting any force. After a 10 s rest, they performed three consecutive isometric MVCs of the shoulder abduction, each one lasting 5 s followed by a 2 min rest. ES was superimposed on the third second of each MVC, when the torque output had plateaued. Excluding the first second of each MVC to disregard any initial sudden movements, the maximum torque of the three repetitions determined the muscle capacity of each participant, hereafter called the pre-MVC. Following 1 min of rest after the last MVC, another ES was delivered at muscle rest (ES_1_).

**Fig. 2:**
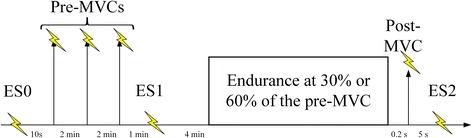
Experimental protocol (: contractions; : ES)

**Fig. 3:**
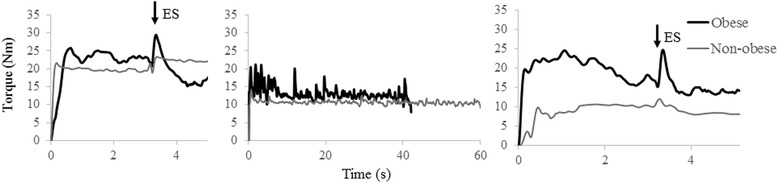
Representative recordings of pre-MVC, endurance trial, and post-MVC for one obese and one non-obese subject

Four minutes later, a sustained isometric endurance task until exhaustion was conducted at either 30 or 60% of the pre-MVC, with each relative target torque (TT) performed during one session. Sessions were separated by at least 2 days and task order was counterbalanced to minimize any residual effects of fatigue. The TTs were set relative to pre-MVCs to minimize the potential confounding effect of supporting a heavier arm in obese individuals, in order to find any physiological differences with obesity. For the endurance task, participants were instructed to ramp up their torque after an alarm and maintain it right above the TT until exhaustion. To attain the target, real-time analog and digital visual feedback was provided. The endurance task was terminated when the mean torque dropped below 10% of the TT and remained as such for at least 1 sec. After a short stop (200 ms) post endurance termination, an ES was superimposed on another 5 s MVC (post-MVC). The 200 ms was considered to standardize the stop time between terminating the endurance task and quickly starting the post-MVC for all participants. After 5 sec, participants received the last ES potentiated at muscle rest (ES_2_). Figure 2 shows the experimental protocol. In Fig. 3, representative recordings of a pre-MVC, endurance, and a post-MVC for one obese and one non-obese subject are illustrated.

### Data reduction

Investigating the effect of BMI-defined obesity on the fatigue mechanism was the primary goal of this study. For that purpose, a measure of central activation ratio (CAR) was used, as calculated by eq. (1), to quantify central fatigue by considering the increment in the torque output by superimposing ES (superimposed twitch) relative to its corresponding MVC. Prior to the fatiguing task, %CAR was averaged over the three pre-MVCs, referred to as pre-CAR. The superimposed ES over the post-MVC determined the post-CAR value after the fatiguing task. The change in %CAR from pre- to post-fatigue has been a common measure in many studies to compare central fatigue (i.e., [37, 48–50]). Percent CAR (%CAR) shows the percent of unfatigued motor units activated voluntarily during maximum contractions. Thus, central fatigue was quantified as a fatigue-induced reduction in activation capability from pre- to post-endurance task (pre- minus post-CAR) [49].

1 $$ \% CAR=\frac{MVC}{MVC+ superimposed\  twitch}\times 100 $$

Peripheral fatigue, on the other hand, was quantified by considering the stimulations of the muscle at rest (i.e., ES_1_ and ES_2_). At muscle rest, the effect of voluntary activation would be eliminated to measure the un-fatigued motor units available to be activated by the ES [46]. ES_0_ was only applied to ensure that all participants had the same level of muscle fatigue when participating in this experiment. Peripheral fatigue was quantified as a decrease of muscle twitch amplitude from pre- to post-task, relative to the pre-task stimulation ((ES_1_ - ES_2_)/ ES_1_).

In a few cases, relatively small increases of the motor unit activation after the fatiguing task result from the effect of synergistic muscles or a lack of maximum effort during the pre-activations. To minimize the likelihood of these errors, these small changes were disregarded resulting in equal pre- and post-twitch amplitudes or zero fatigue quantification.

In addition to the mechanism of fatigue, the physical manifestation of fatigue development was evaluated by endurance time, torque fluctuation, and torque loss. Torque loss was quantified as the percent of change from pre- to post-MVC relative to the pre-MVC. In addition, rate of torque loss per second over the endurance time ((pre- minus post-MVCs)/endurance) was calculated. Torque fluctuation were calculated by the coefficient of variation (CV = standard deviation/mean) for each 5 sec non-overlapping window during the endurance task. The average (TF_a_) and linear rate of torque fluctuation (TF_r_) were then considered. To test the fatigue state of the muscle, the root-mean-square (RMS) and median-power-frequency (MPF) of the EMG power spectrum were calculated over 0.125 s windows with 0.0625 s overlaps during the endurance effort. Built-in filters from Delsys EMGworks Acquisition software Version 4.1.1 were used to process the EMG data in real-time. The slopes of the linear regression for RMS and MPF were calculated and used for EMG temporal behavior. The joint changes in the EMG measures were used to indicate the muscle fatigue- versus force-induced states [51]. Based on this analysis, four states of recovery, force increasing, force decreasing and fatigue can be recognized with considering the slopes of RMS and MPF changes simultaneously.

Finally, relative target loads (i. e., 30 or 60%MVC) were converted to the absolute target loads (in Nm) that each participant exerted at in each session. The effect of absolute TT on the endurance time was then modeled. After testing linear, logarithmic, polynomial, power, and hyperbolic curves, all with the same number of parameters, the best fit curve (as determined by the highest R^2^) was achieved by a negative exponential curve in the form of *Endurance time* = *ae* ^−*b* ∗ *TT*^, where *a* is the endurance time at zero TT, and *b* is the exponential decay rate.

### Statistical analysis

After extracting all dependent variables, separate analyses of covariance (ANCOVA) were conducted to assess differences between the obese and non-obese groups controlling for age and gender. All three assumptions of normality, homogeneity of variance, and independency of residual errors were checked by using Shapiro-Wilk test, Leven’s and Durbin-Watson tests, respectively, and by visual inspections. For the pre- and post-MVC and TF_a_ data, natural log transformation was used to meet the assumptions. Also, square root transformation and Box-Cox transformation with λ = 3 were used for endurance time, and post-CAR, respectively. Non-parametric Mann-Whitney U tests were applied for torque loss, rate of torque loss, TF_r_, RMS, MPF, central and peripheral fatigue, where data transformation could not validate the assumptions. For the central and peripheral fatigue measures, the electrical stimulation intensity was included in the ANCOVA model as a covariate and dominant hand was included as a blocking variable. This was done to control for the effect of handedness and reduce any systematic noise in the error term. Independent samples *t*-tests were performed to compare endurance time model parameters between the obese and non-obese groups. All statistical analyses were performed in SPSS Version 22 (IBM Corporation) with the level of significant set at α = 0.05.

## Results

Obese individuals tolerated a higher (*p* = 0.001) current intensity with an average (SD) of 36.3 (10.35) mA compared to 29.75 (7.05) mA for the non-obese group. For the dependent measures, means, standard deviations (SD) are summarized in Table 2 for each load and obesity group. Also, *p*-values of the statistical analyses between the obese and non-obese groups are reported in Table 2.

**Table 2:** Results are presented as mean(SD)

Measures	Normal (*n* = 22)		Obese (*n* = 17)		*p*
	30%	60%	30%	60%	
Pre-MVC (Nm)	24.8(9.3)	25.7(9.6)	28.0(13.4)	30.7(13.8)	.955
Endurance (s)	64.6(26.9)	24.6(12.8)	56.3(25.6)	24.2(18.2)	.266
Post-MVC (Nm)	17.3(6.9)	20.5(9.8)	17.7(9.2)	24.8(12.7)	.483
Pre-CAR (%)	85.0(6.2)	85.1(6.6)	86.4(5.2)	90.3(5.3)	.018*
Post-CAR (%)	80.3(12.1)	82.9(9.9)	73.2(15.6)	79.9(9.0)	.068
Central fatigue (%)	7.2(9.6)	4.3(5.4)	14.2(12.8)	11.3(8.4)	.001*
Peripheral fatigue (%)	26.6(29.0)	21.4(24.0)	17.7(30.2)	13.2(25.8)	.061
RMS Slope(×10^−6^)	−.58(1.2)	−.74(9.7)	.23(.68)	2.18(6.4)	.019*
MPF Slope	−.48(.37)	−.91(.78)	−.58(.31)	−.75(1.72)	.496
TF_r_ (1/s)	.03(.04)	.08(.10)	.04(.07)	.08(.09)	.232
TF_a_ (Nm)	.17(.02)	.15(.07)	.17(.05)	.15(.05)	.911
Torque loss (% Pre-MVC)	29.8(17.4)	20.4(16.2)	38.0(18.1)	19.8(16.5)	.304
Torque loss rate (Nm/s)	.14(.12)	.29(.32)	.21(.16)	.49(.91)	.081

Note: ANCOVA modeling required a natural log transformation of pre- and post-MVC. Square root transformation was used for endurance time and a Box-cox transformation with λ = 3 was applied to post-CAR

Significant *p*-values are bolded and marked with*

### Central vs. peripheral fatigue with obesity

A comparable muscle twitch was observed at the beginning of the experiment with an average (SD) ES_0_ of 0.65 (0.79) Nm for the non-obese and 0.63 (0.66) Nm for the obese group. On average, obese individuals had a higher pre-CAR compared to the non-obese individuals (88.3% compared to 85.1%, respectively). However, the post-fatigue activation ability of the obese group decreased ~11.7% versus an ~3.5% reduction for the non-obese group resulted in the post-CAR values of 76.6 and 81.6%, respectively. Results show a significant between group difference in the pre-CAR (*p* = 0.018) and a trend toward significant difference in the post-CAR (*p* = 0.068). A higher central fatigue (*p* = 0.001) for individuals who are obese compared to non-obese individuals was observed. Contrary to the effect of obesity on central fatigue, a trend toward reduced peripheral fatigue (*p* = 0.06) was observed for the obese group compared to the non-obese group by calculating the percent change from ES_1_ and ES_2_ before and after the endurance task relative to the ES_1_ (24.0 (26.5) % for non-obese vs. 15.4 (27.8) for obese). Obese participants had on average 8.6% less decrease on the relative number of available motor units when getting fatigued. A significantly higher positive trend of RMS change (*p* = 0.019) with obesity was observed. The slope of MPF was comparable and negative for both groups.

### Physical fatigue manifestations with obesity

Comparable pre- and post-MVC, endurance time, and torque loss were found between the obese and non-obese groups. However, normalizing the torque loss to the endurance time, to calculate the torque loss rate per second, resulted in a tendency toward a significant effect of obesity (*p* = 0.081). Obese individuals had on average a 14% higher rate of torque loss per second compared to non-obese participants.

**Fig. 4:**
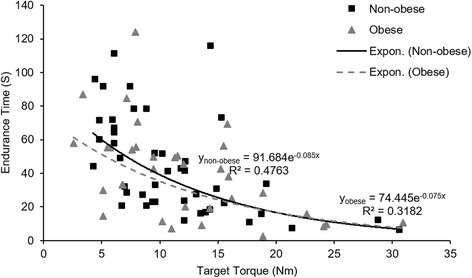
Endurance time (s) and absolute target torque (Nm) relationship

The relationship between endurance time and absolute TT was assessed after converting the relative to absolute TT for each subject. The best fit curve is shown in Fig. 4 with R^2^ = 0.48 and 0.32 for the non-obese and obese groups, respectively. Based on the aforementioned R^2^ values, both models fail to fully explain the variability in endurance time. However, two independent samples *t*-tests indicated significant differences of model parameters *a* (*p* < 0.001) and *b* (*p* = 0.009) between the two groups. Endurance time estimation at very low TT (asymptotic to zero) indicated that the tolerance of the obese individuals is ~19% of the tolerance of the non-obese individuals. However, exponential decay rate for the obese individuals is ~12% of the decay rate for the non-obese group when moving toward higher loads.

## Discussion

The increased central fatigue with obesity found in this experiment supported the first hypothesis. The second hypothesis was partially supported by the results. Obesity-related impairment of the middle deltoid muscle capacity was evident only in a trend toward a higher rate of torque loss.

### Central vs. peripheral fatigue with obesity

The joint analysis of EMG measures [51], the positive trend of RMS and negative trend of MPF change, indicate a fatigued state for the obese individuals. In contrast, the negative trends of RMS and MPF change indicate a force decreasing state for the non-obese group, which suggests that they stopped the endurance task prior to a fatigued state. The fatigue state of the middle deltoid of the obese individuals was diagnosed as the result of central rather than peripheral fatigue in this study. This was found from a greater reduction from pre- to post- CAR, which indicated a greater reduction in the ability of the obese group to voluntarily activate their available motor units once they fatigued. Similarly, reduced motor unit activation with obesity was reported for knee extensor [29, 30] and ankle dorsiflexor [31] muscles suggesting a greater role of central fatigue with obesity for the lower extremities. Central fatigue impairment with obesity reported for the lower extremities was verified for the middle deltoid muscle in this study.

Quantification of the role of central fatigue in torque loss provides a useful metric for between-group comparison. Previously, 16% central fatigue, calculated as a CAR drop from 0.94 –0.78, during a fatiguing task of ankle dorsiflexor resulted in a 78% torque loss for normal weight participants [45]. Therefore, a 20% contribution of central fatigue in the muscle fatigue development was estimated. In this study, the ~12% CAR reduction caused an ~29% torque loss, leading to an estimation of ~42% contribution of central fatigue for the obese individuals. For the non-obese group, only ~14% (~3.5% central fatigue of the ~25% torque loss) of the muscle fatigue was due to central fatigue. An ~3 times greater contribution of central fatigue for the obese individuals compared to non-obese individuals could be a concern especially during longer exertions, where a higher contribution of central fatigue has been suggested [34].

Contrary to having a higher central fatigue impairment, obese individuals had a trend toward a lower peripheral fatigue compared to their non-obese counterparts. Keeping the ES intensity constant, a greater decrease in the ES amplitude from pre- to post-task relative to pre- task was observed for the non-obese group. Central fatigue impairment might cause a faster task termination for the obese individuals before they reach to a comparable peripheral fatigue. A comparable peripheral fatigue would be expected for the obese group if central fatigue did not hinder the task continuation.

### Physical fatigue manifestations with obesity

An equivalent endurance time at both 30 and 60% MVC was observed between obese and non-obese groups, which is consistent with comparable times to task failure at relative TTs reported for the quadriceps muscle [45]. However, when considering absolute targets that participants tolerated, a shorter endurance time of obese individuals compared to non-obese individuals was more evident for lower absolute TT (i.e., less than 15 Nm; Fig. 4), where type I muscle fibers are mainly engaged in the force retention. This is consistent with a reduced isometric shoulder muscle endurance at a low absolute TT (9 Nm) reported by Cavuoto and Nussbaum [17]. They also found a greater shoulder muscle torque loss, which suggested an obesity-related impairment of shoulder muscle functional capacity at low loads. At high absolute TTs or short endurance times, any obesity-related differences might not have a chance to manifest.

Impaired middle deltoid muscle capacity was not evident in the torque loss and torque fluctuation or its rate of change over the endurance trial at either low or high relative TT for young healthy participants. Similar results were reported with an isometric ankle dorsiflexion endurance task at 60% MVC [31]. In addition, no effect of obesity on joint stability during fatiguing elbow [52] and shoulder [17, 53] flexion tasks were observed previously. Likewise, upper extremity neuromuscular control between non-obese and obese groups at relative TT of 15 and 40% MVC found to be comparable in another study [54].

### Limitations

Due to some limitations, the results of this study should be interpreted with caution. First, the BMI and age recruitment criteria in this study were limited to obesity classes Ι and Π (30 < BMI < 40 kg/m^2^) and younger adults, respectively. Extremely obese individuals (BMI > 40 kg/m^2^) were excluded from this study since they only consist ~6% of the population [55]. Second, there are some limitations associated with using ES to quantify central and peripheral fatigue. Detecting small twitches with regards to the background noises of maximal exertions was challenging. To minimize errors, a higher resolution (1024 Hz data collection frequency), averaging technique over pre-MVCs, and a custom-written Matlab code and visual inspection were used to identify and confirm the twitches, however this might have introduced some error in the results. Surface ES might not have activated those motor units deep in the muscle [56]. Even after identifying central fatigue with obesity using ES, the underlying reasons of this impairment, including impaired signal generation or propagation, incomplete motor unit activation or recruitment, or lack of motivation, are not known. Further investigations on the possible reasons for central fatigue impairment with obesity are needed to supplement this research. Moreover, although, participants were firmly secured in a fixed posture to isolate the middle deltoid in shoulder abduction, the effect of possible excitation of antagonist muscles was overlooked in the analysis. Also, the comparisons in this study were based on relative TTs, whereas, in most settings absolute loads are required regardless of individuals’ capacities. It is unclear whether the same results would have been observed under the same absolute TT for both groups. Lastly, cautious interpretation of the results is advised due to the transformation of variables to meet the normality assumptions.

## Conclusion

Overall, the results of this study suggest that a meaningful difference of physical fatigue manifestations of the middle deltoid muscle is unlikely unless at higher classes of obesity (BMI > 35 kg/m^2^) [32] or when interacting with the effects of aging [17]. However, a greater contribution of central fatigue was observed with obesity during the sustained endurance tasks. This suggests that the faster fatigue development with obesity observed in many other studies likely originated in the central elements rather than the peripheral factors for young healthy obese individuals. The current signs of central fatigue could lead to impaired motor performance, especially for extremely obese or older obese individuals.

## Abbreviations

ANCOVA: Analysis of covariance
BF: Body fat
BMI: Body mass index
CAR: Central activation ratio
CNS: Central nervous system
CV: Coefficient of variation
EMG: Electromyography
ES: Electrical stimulation
MPF: Median-power-frequency
MVC: Maximum voluntary contraction
RMS: Root-mean-square
TF: Torque fluctuation
TT: Target torque
WMSDs: Work-related musculoskeletal disorders
